# The Time–Concentration–Mortality Responses of Western Flower Thrips, *Frankliniella occidentalis*, to the Synergistic Interaction of Entomopathogenic Fungus *Metarhizium flavoviride*, Insecticides, and Diatomaceous Earth

**DOI:** 10.3390/insects11020093

**Published:** 2020-02-01

**Authors:** Wenchao Ge, Guangzu Du, Limin Zhang, Zhengyue Li, Guanli Xiao, Bin Chen

**Affiliations:** 1 State Key Laboratory for Conservation and Utilization of Bio-Resources in Yunnan, College of Plant Protection, Yunnan Agricultural University, Kunming 650201, China; gewenchao95@163.com (W.G.); duguangzu1986@163.com (G.D.); limin0789@126.com (L.Z.); kmlizhengyue@163.com (Z.L.); 2 International Cooperation and exchange Department, Yunnan Agricultural University, Kunming 650201, China; 3 College of Agronomy and Biotechnology, Yunnan Agricultural University, Kunming 650201, China

**Keywords:** *Frankliniella occidentalis*, *Metarhizium flavoviride*, insecticides, diatomaceous earth, synergism, time–concentration–mortality modelling

## Abstract

Western flower thrips (WFT), *Frankliniella occidentalis* (Pergande), is a highly invasive pest which is harmful to many cash crops globally and resistant to various insecticides. Entomopathogenic fungi (EPF), as biological control agents, have demonstrated a good control effect on WFT. The aim of this study was to evaluate the synergistic and pathogenicity efficacy of the fungal strain *Metarhizium flavoviride* WSWL51721 when distributed with diatomaceous earth (DE) and the active ingredient imidacloprid using four bioassay methods against adult and second instar larvae of WFT. The data of the four bioassays have been fitted to the time–concentration–mortality (TCM) model. The corrected mortality ranges of WFT adults were 75–100%, 82.69–100%, 78.85–100%, and 92.31–100%, and the corrected mortality ranges of WFT second instar larvae were 72.22–100%, 85.19–100%, 77.77–100%, and 100% in the four bioassays at concentrations of 1.2 × 10^6^ to 1.2 × 10^8^ conidia/mL, respectively. At 1.2 × 10^8^ conidia/mL, assays 2 (*M. flavoviride* with DE), 3 (*M. flavoviride* with imidacloprid), and 4 (*M. flavoviride* with DE and imidacloprid) had the shortest median lethal time (LT_50_), compared with that of assay 1 (*M. flavoviride* alone) for adults at 2.26 d, 2.06 d, and 1.53 d, and second instar larvae at 2.45 d, 1.70 d, and 1.41 d, respectively. The median lethal concentration (LC_50_) in the four bioassays decreased within 3–10 days of inoculation. On the third day, it was found that the lowest median lethal concentrations in assays 2, 3, and 4 were 1.58 × 10^7^, 1.13 × 10^7^, and 3.39 × 10^6^ conidia/mL, respectively, which were significantly different from that in assay 1 for the adults. For the second instar larvae, assays 2, 3, and 4 also had the lowest lethal concentrations and were significantly different from those of assay 1. There were significant differences in sporulation between adults and second instar larvae under the four bioassays. Our results indicate that assays 2 (*M. flavoviride* with DE), 3 (*M. flavoviride* with imidacloprid), and 4 (*M. flavoviride* with DE and imidacloprid) demonstrate synergistic effects on the control of both adult and second instar larvae of WFT under laboratory conditions.

## 1. Introduction

Western flower thrips (WFT), *Frankliniella occidentalis* (Pergande) (Thysanoptera: Thripidae), is an invasive pest in agroforestry worldwide, impacting many economically important greenhouse-grown crops, such as ornamental flowers, vegetables, fruits, and other various plants through their piercing-sucking mouthparts, causing direct feeding damage and transmission of several destructive plant viruses which have caused significant economic losses in many open-field and greenhouse crops [1,2,3,4,5,6].

Currently, due to the wide suitable area for WFT, they pose a huge threat to agriculture and forestry crops. Therefore, chemical insecticides have been mainly applied to prevent and control WFT; however, due to the unreasonable and intensive use of various insecticides, WFT have become resistant to various insecticides, such as organophosphorus, organochlorine, carbamate, pyrethroids, and spinosad. Thus, the management of WFT has become a complex task [2,7,8,9,10]. Moreover, due to the high toxicity of chemical insecticides, problems with large residual amounts of chemicals and a long residual period in marketable cash crops, contamination of the environment, threats to human health, toxicity to beneficial non-target organisms, species displacement, and destruction of IPM systems [3,11,12,13,14], finding reliable biological control methods that can protect the ecological environment and control the population of WFT effectively and continuously, as well as being compatible with other components, has become an important area of research for the integrated control of WFT [3,13,15].

Entomopathogenic fungi (EPF) are biological control agents with insecticidal activities against a variety of agricultural insect pests worldwide [16,17,18,19]. Most importantly, they do not pollute the environment, are harmless to humans and animals, have an ecologically wide distribution, and target a variety of host species, demonstrating a broad spectrum of insecticidal effectiveness and strong pathogenicity against many insect pests in the agricultural field. Consequently, they have been widely used and studied throughout the world [17]. There are seven species of entomopathogenic fungi which have been successfully applied for the control of WFT, including *Lecanicillium lecanii* (Zimmermann) [20], *Beauveria bassiana* (Balsamo) Vuillemin (Hypocreales: Ascomycota) [6,21,22,23,24], *Metarhizium anisopliae* (Metschnikoff) Sorokin (Hyphomycetes: Deuteromycotina) [3,9,10,12], *Metarhizium brunneum* (Metschnikoff) Sorokin [25], *Metarhizium flavoviride* (Gams and Rozsypal) (syn. *Metarhizium anisopliae* var. *Acridum*, pro parte) (Hyphomycetes: Deuteromycotina) [26], *Neozygites parvispora* (Zygomycotina: Entomophthorales) [27], and *Isaria* (*Paecilomyces*) *fumosorosea* (Wize) Brown and Smith [6,10], among which the fungal pathogens *B. bassiana* and *M. anisopliae* have been the most studied for the control of WFT. In addition, as the essential oils (EOs) of botanical insecticides have similar control efficacies on pests as EPF, there have also been many studies on EOs in controlling WFT [28,29].

Diatomaceous earth (DE) belongs to inert dusts, a large class of substances with stable properties which do not easily produce chemical reactions. DE has a good ability to absorb esters, and so it is a potential natural insecticide [30,31,32]. There are numerous reports, both domestic and foreign, which have indicated that DE has broad prospects for controlling a wide range of store-product pests, such as *Tribolium confusum* (J du Val), *Tribolium castaneum* (Herbst), *Acanthoscelides obtectus* (Say), *Cryptolestes ferrugineus* (Stephens), *Sitophilus granarius* (L.), and *Rhyzopertha dominica* (F.) [15,33,34,35,36], as well as a variety of agricultural pests, such as *Solenopsis invicta* (Buren), *Gryllidae* (Bolívar), and *Leptinotarsa decemlineata* (Say) [37,38]. The insecticidal principle of DE is mainly to adsorb the cuticular lipids from the targeted insects; the tiny DE particles are trapped by the exposed cuticle surface of an insect, which causes various kinds of physical or mechanical damage and loosens the cuticle, causing loss of water and resulting in death [39,40,41,42,43].

EPF have been demonstrated to control different insect pests, but have a relatively slow effect compared with chemical insecticides, as all fungal agents have a latent period in their host after infecting the target pest [44,45,46,47]. In addition, a potential disadvantage of EPF is their relatively short shelf life, compared to conventional chemical insecticides [6]. Further, when the pest population density is high, it is difficult to control the pest density below an economic threshold with only the use of a single fungal agent [12]. Therefore, combining fungal biocontrol agents with adjuvants and other insecticides with good compatibility is an effective and reliable solution to improve the pest control effect [15,18,32,39,48]. Numerous studies have reported that the combination of EPF with insecticides [44,48,49,50], DE [34,39,51,52], and with insecticides and DE have significantly improved their pest control efficacies [15,32,33,35]. For example, Nian et al. [49] demonstrated that combining *I. fumosorosea* with sub-lethal doses of the insecticides beta-cypermethrin and *bacillus thuringiensis* caused synergistic effects against *Plutella xylostella* larvae under laboratory conditions. In another study, Wakil et al. [32] demonstrated that combining *B. bassiana* with the neonicotinoid insecticide thiamethoxam and DE led to synergistic effects against *Rhyzopertha dominica* under laboratory conditions. At present, there are two main strategies for the biological control of WFT: (1) EPF combined with predatory mites [12,13,20,53]; and (2) EPF combined with insecticides [3,6,9,10]. For example, Wu et al. [13] demonstrated that the combination of *B. bassiana* and the predatory mite *Neoseiulus barkeri* improved WFT control in greenhouse-grown cucumbers; similarly, Jacobson et al. [53] showed that the simultaneous use of *Amblyseius cucumeris* and *B. bassiana* improved WFT control in cucumbers. In addition, Ansari et al. [9] demonstrated that *M. anisopliae* combined with sub-lethal doses of conventional insecticides (imidacloprid and fipronil) had a better control effect on WFT than the individual fungal biocontrol agents.

However, the synergistic effects between *M. flavoviride* and insecticides and DE against WFT have not been reported. Therefore, in this study, we first evaluated the compatibility of three commonly used chemical insecticides with *M. flavoviride*, and the best compatible insecticide was selected to combine treatment with *M. flavoviride* and DE, which was analyzed through the use of the complex but robust method of time–concentration–mortality responses, instead of simple concentration–mortality responses, to discern the virulence of different bioassays in adults and second instar larvae of WFT under laboratory conditions.

## 2. Materials and Methods

### 2.1. Rearing Protocols for WFT

A number of WFT (*Frankliniella occidentalis*) were collected from rose petals in greenhouses of the Dounan Flower Planting Base in Kunming, Yunnan province (24°90′ N, 102°79′ E), in 2018. WFT were maintained as described by Ansari et al. [9], with slight modifications. Briefly, thrips demes were continuously reared on sterilized kidney bean pods (*Phaseolus vulgaris* L.) in tissue culture bottles (9.1 cm in height, 6.9 cm in diameter; Xuzhou Hualian Glass Products Co., Ltd., China) and covered with a fine mesh to allow for ventilation. These bottles were kept at 25 ± 1 ℃, 65 ± 5% RH, and with a L 14 h:D 10 h photoperiod in growth chambers (RG-300, Guangzhou Kenton Apparatus Co., Ltd., China). Coeval healthy thrips cohorts were obtained by rearing adults on fresh and healthy kidney bean pods for oviposition. After three days, the eggs laid on the pods were transferred to fresh ventilated tissue culture bottles. The first instar larvae hatched from the eggs 2 days later. The second instar larvae were collected three days post-eclosion. The second instar larvae and adults were collected from the tissue culture bottles for experimental use.

### 2.2. Fungal Strains and Preparation

The fungal strain WSWL51721 of *Metarhizium flavoviride* was originally isolated from a naturally infected cadaver of an unknown *Coleoptera* adult in the Xishuangbanna National Nature Reserve in Yunnan Province in 2017 and maintained at 4 ℃ in the Insect Fungi Laboratory of the College of Plant Protection, Yunnan Agricultural University. The strain was cultured on Sabouraud Dextrose Agar (SDA) medium at 25 ± 1 ℃, 75% RH, and under conditions of 16 h light/8 h dark. After seven days, a large number of conidia were harvested from the Petri plates by gently scraping the surface of the SDA medium, which were suspended in sterile aqueous 0.05% TWEEN^®^ 80 (Beijing Solaibao Technology Co. Ltd., China). Conidial suspensions were quantified using a Neubauer Hemocytometer (Shanghai Anxin Optical Instrument Manufacturing Co., Ltd., China) and the experimental concentration was adjusted to 1.2 × 10^8^ conidia/mL using sterile 0.05% TWEEN^®^ 80. Conidial suspensions were diluted to 1.2 × 10^6^ conidia/mL for subsequent experiments. The viability of the conidia was assessed according to the method of Goettel and Inglis, demonstrating that the percentage germination was >90% [54].

### 2.3. DE and Insecticide Formulations

The diatomaceous earth (DE) formulation used in this experiment was Puliangtai^®^ (Shanxi Keli Science and Technology Co., Ltd., China), which contained 85% amorphous silicon dioxide. Three insecticides commonly used in the control of WFT were selected: The commercial emulsifiable concentrate of Abamectin, containing 18 g/L of active ingredient (Henan Yongguan Qiaodi Agricultural Technology Co., Ltd., China); the commercial wettable granule formulation of Imidacloprid, containing 700 g/L of active ingredient (Bayer CropScience Limited, China); and the commercial wettable powder formulation of Buprofezin, containing 250 g/L of active ingredient (Jiangsu Yangzhou Suling Pesticide Chemical Co., Ltd., China) were used in the experiments.

### 2.4. Radial Hyphal Growth Test in the Presence of Insecticides

Imidacloprid, buprofezin, and avermectin were tested at three dose rates to evaluate their inhibitory effects on the radial growth of *M. flavoviride*. The fungal strain culture was performed as described in Section 2.2. After autoclaving, when the SDA medium temperature dropped to 45 ℃, but was not yet solidified and would not affect the pesticide activity [19], each different concentration of pesticide prepared with sterile distilled water was added to the culture medium for homogenization, and then poured into the Petri dishes for solidification. Next, 4 mm diameter fungal strain cores were taken from the *M. flavoviride* cultures and placed upside down in the middle of Petri dishes containing SDA medium with 0 (control), 10, 50, and 100 ppm of imidacloprid, buprofezin, and avermectin solution, respectively. The potential inhibitory effects of the insecticides on radial hyphal growth were determined as described by Russell et al. [48]. Briefly, surface radial growth was measured for 10 days on alternate days. Two radii at right angles to each other drawn on the bottom of each dish were measured using an Electronic Digital Caliper (Qingdao Meijite Precision Tools Co., Ltd., China). Each treatment was conducted three times.

### 2.5. Conidia Production Measure in vitro in the Presence of Insecticides

The fungal strain conidia production was measured using a slight modification of the technique described by Russell et al. [48]. Briefly, imidacloprid, buprofezin, and avermectin were incorporated into SDA medium at concentrations of 0 (control), 10, 50, and 100 ppm. Six replicate Petri dishes per treatment were inoculated with 4 mm diameter cores of fungal strain from the *M. flavoviride* cultures, then maintained at 25 ± 1 ℃ with a 16 h light (L):8 h dark (D) photoperiod. After ten days, three 4 mm diameter cores, from the center point of each colony to ^1^/_2_ of the edge distance, were taken from each Petri dish and added separately to 2 ml microcentrifuge tubes containing 1 ml of sterile 0.05% TWEEN^®^ 80. Conidia suspensions from each core were quantified using a Neubauer Hemocytometer to estimate conidia per cm^2^. The experiment was replicated three times.

### 2.6. Bioassays

In the bioassays, imidacloprid was selected and applied at 50 ppm, based on radial hyphal growth and conidia production of fungal strain in the presence of insecticides according to Section 2.4 and Section 2.5, while diatomaceous earth was tested at 200 ppm. First, the virulence effects of diatomaceous earth, imidacloprid, and diatomaceous earth combined with imidacloprid were examined for the control of adults, and second instar larvae of WFT (*F. occidentalis*) were tested. Secondly, the fungal strain was tested at the two concentrations of 1.2 × 10^6^ and 1.2 × 10^8^ conidia/mL. Imidacloprid and diatomaceous earth were tested at 50 ppm and 200 ppm, respectively. Four bioassays, as described in Table 1, were performed using immersion for the adults and the second instar larvae of WFT. For inoculation, the adults and second instar larvae were individually immersed in various concentrations of fungus alone, as well as fungus + insecticide, fungus + diatomaceous earth, and fungus + diatomaceous earth + insecticide combinations for 10 s. Excess suspension was then removed by filter paper, and 20 adults and second instar larvae were carefully transferred to a sterile glass insect tube (10.0 cm height, 3.0 cm diameter) using a fine brush; fresh kidney bean pods served as a food source. All treated adults and second instar larvae were kept in glass insect tubes (10.0 cm height, 3.0 cm diameter) and sealed with 200 mesh gauze and rubber bands to prevent the WFT from escaping during the treatments.

The test insects were incubated at 25 ± 1 ℃ and 65 ± 5% RH with a L 14 h:D 10 h photoperiod in growth chambers. The experiment was repeated three times. The mortality of adults and second instar larvae were observed daily for 10 days, and fresh untreated pods were added every two days. Dead adults and second instar larvae were collected from all treatment groups, the WFT carcasses were surface disinfected with 70% ethanol for 2–3 min, followed by three washings in sterile distilled water, and were placed on moist sterilized filter paper in the same Petri dishes, sealed with parafilm, and incubated at 25 ± 1 °C and 75 ± 5% RH for 3–5 days to promote fungal hyphal growth on the surface of the carcass and to further confirm that death was caused by mycosis using a light microscope (LEICA-M205FA, Leica, Germany).

### 2.7. Quantification of Spore Production on Mycotized WFT Carcasses

All *M. flavoviride*-sporulating adults and second instar larvae from Section 2.6 in the four bioassays were cultured in petri dishes lined with moist, sterilized filter paper for 12 days. Fungal growth on the carcass surface was observed every day in order to ensure that fungal sporulation had occurred on the surface of all WFT carcasses [48]. Twenty adults and second instar larvae from each of the above treatments were separately transferred to a 1.5 mL sterile centrifuge tube supplemented with 1 mL of sterile 0.05% TWEEN^®^ 80, followed by shaking on an orbital shaker for 10 min [55]. The amount of conidia on each insect was estimated by counting the total number of conidia using a Neubauer Hemocytometer. Each treatment was repeated three times.

### 2.8. Statistical Analysis

Radial hyphal growth and conidia production of *M. flavoviride* in the presence of insecticides, as well as the data of number of conidia on the carcass, were analyzed by one-way ANOVA. Means were compared using Tukey’s HSD test at a 5% level of significance. All data were analyzed using the Data Processing System (DPS) software, version 14.0 [56], as well as the SPSS statistical software, version 20.0.

As the Time–Concentration–Mortality (TCM) model can combine time and dose effects into a single model, the interactions of time and dose effects can be examined to reflect the integrity and objectivity of bioassay data. Therefore, the data from the different bioassays (i.e., 1–4) were analyzed using the TCM model to determine the interactions between *M. flavoviride* and the adults and second instar larvae of WFT [57,58,59]. Briefly, the adults and second instar larvae of WFT mortality (*q_ij_*), called the conditional mortality probability, caused by a given conidial suspension concentration of *M. flavoviride* (*d_i_*) at specific time intervals [*t*_*j*−1_, *t_j_*] (i.e., day 1, day 2, …, day 10 after immersion treatment) were corrected with background mortalities (mortality was only treated with sterilized 0.05% TWEEN^®^ 80) observed from the controls of each assay. This was then fitted to the conditional TCM model *q_ij_* = 1 − exp[−exp(*γ_j_* + *β* log10(*d_i_*))] by approaching a maximum likelihood equation $\overset{J}{\underset{j=1}{\prod }}\overset{I}{\underset{i}{\prod }}q_{ij}^{r_{ij}}{\left(1-q_{ij}\right)}^{n_{ij-r_{ij}}}$, where *n_ij_* represents the number of adults and second instar larvae of WFT surviving at time *t*_*j*−1_ after an inoculation dose *d_i_,* and *r_ij_* represents the number of adults and second instar larvae of WFT caused by *M. flavoviride* infection by the time interval [*t*_*j*−1_, *t_j_*]. This conditional modelling of the *β* and *γ_j_* estimates were used to determine the cumulative TCM relationships in the form of *p_ij_* = 1 − exp[−exp(*τ_j_* + *β*log10(*d_i_*))], which were obtained with the cumulative time effect of *τ_j_* = ln($\overset{j}{\underset{k=1}{\sum }}e^{\gamma k}$). The parameter estimates of *β* and *τ_j_*, as well as their variances and covariances, were finally used to calculate the lethal concentrations (LC), including LC_50_ and LC_90_ (the conidial concentrations of *M. flavoviride* causing 50 or 90% of the adults and second instar larvae of WFT to die, respectively) as a function of time after immersion treatment and the lethal times (LT), including LT_50_ and LT_90_ (the lethal time for a given concentration of *M. flavoviride* to cause 50 or 90% mortality of the adults and second instar larvae of WFT) as a function of the conidial concentration.

## 3. Results

### 3.1. Radial Hyphal Growth in the Presence of Insecticides

**Figure 1:**
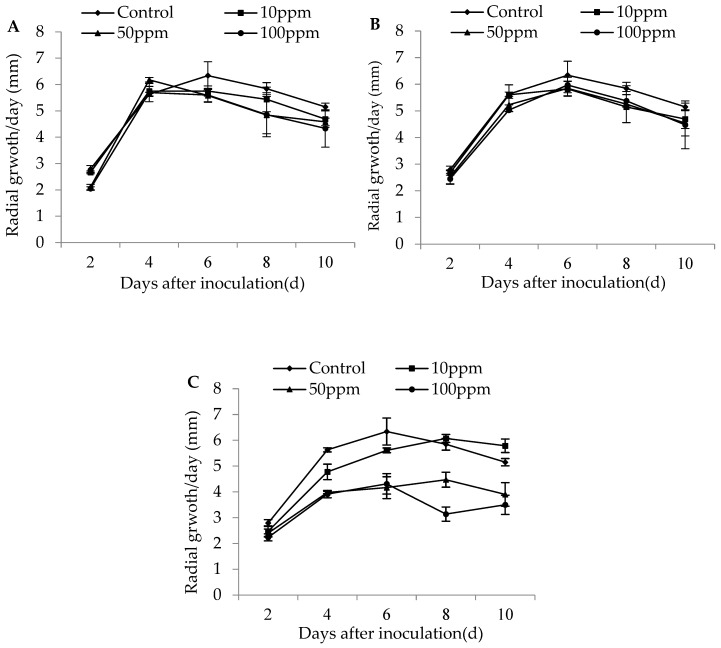
Radial growth rate (mm/day ± SE) of *M. flavoviride* on SDA added with three insecticides: (**A**) 10, 50, and 100 ppm Imidacloprid; (**B**) 10, 50, and 100 ppm Buprofezin; and (**C**) 10, 50, and 100 ppm Abamectin.

The colony growth of *M. flavoviride* after 2 days’ inoculation on SDA plates was significantly affected by different dose rates of imidacloprid (D2: F_3,11_ = 16.19, *p* = 0.0009 < 0.01), but had no significant effect on the colony growth of the strain during other inoculation days (D4: F_3,11_ = 1.51, *p* = 0.29; D6: F_3,11_ = 0.49, *p* = 0.69; D8: F_3,11_ = 0.65, *p* = 0.61; D10: F_3,11_ = 0.72, *p* = 0.57). No significant effect was found for the dose rates of buprofezin on the colony growth of *M. flavoviride* on SDA plates for any inoculation day (D2: F_3,11_ = 0.69, *p* = 0.58; D4: F_3,11_ = 1.62, *p* = 0.26; D6: F_3,11_ = 0.44, *p* = 0.73; D8: F_3,11_ = 0.41, *p* = 0.75; D10: F_3,11_ = 0.32, *p* = 0.81). The colony growth of *M. flavoviride* on SDA plates at 4, 6, 8, and 10 days of inoculation was significantly affected by different dose rates of abamectin (D2: F_3,11_ = 2.09, *p* = 0.18; D4: F_3,11_ = 21.90, *p* = 0.0003 < 0.01; D6: F_3,11_ = 7.11, *p* = 0.01; D8: F_3,11_ = 31.41, *p* = 0.0001 < 0.01; D10: F_3,11_ = 10.45, *p* = 0.004 < 0.01). The three different dose rates of imidacloprid and buprofezin did not significantly affect the colony growth of *M. flavoviride* inoculated on SDA plates, indicating that the two insecticides had good compatibility with *M. flavoviride*. The differences of the daily growth rate of colony diameter (in mm) for *M. flavoviride* by adding low (10 ppm) and medium (50 ppm) dose rates of imidacloprid were not significantly different from those in the control, and the highest radial growths were observed to be 5.76 ± 0.19 mm and 6.18 ± 0.09 mm after days 6 and 4 of incubation, respectively. The differences in radial hyphal growth of *M. flavoviride* by adding low and medium dose rates of buprofezin (10 and 50 ppm, respectively) were not significantly different from those of the control, where the highest radial growths were 5.83 ± 0.28 mm and 5.85 ± 0.27 mm after 6 days of incubation (Figure 1).

### 3.2. Conidia Production Measure in vitro in the Presence of Insecticides

**Figure 2:**
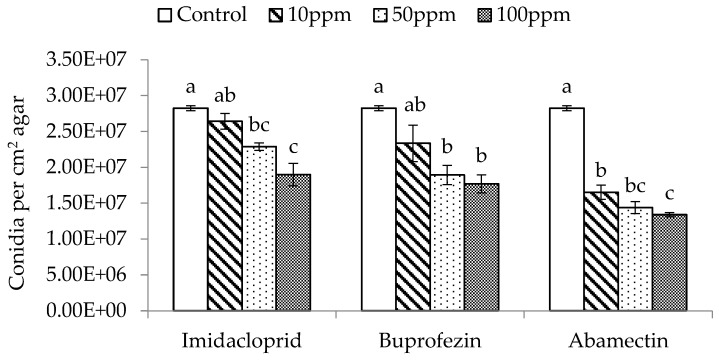
Mean (± SE) conidia production per cm^2^ on SDA with added insecticides Imidacloprid, Buprofezin, and Abamectin at 10, 50, and 100 ppm. The same letters above the bar indicate no significant difference (Tukey’s HSD at *p* = 0.05).

The three different dose rates (10, 50, and 100 ppm) of imidacloprid, buprofezin, and abamectin significantly affected the spore production of *M. flavoviride* inoculated on SDA plates, compared with the control (F_3,11_ = 16.60, *p* = 0.0009 < 0.01; F_3,11_ = 9.25, *p* = 0.0056 < 0.01; F_3,11_ = 99.71, *p* = 0.0001 < 0.01). The spore production of *M. flavoviride* on SDA plates decreased with an increase of dose rate in all three insecticides, and was significantly affected by the three insecticides at low-, medium-, and high-dose rates (10, 50, and 100 ppm) as well (Figure 2). The spore production of *M. flavoviride* on SDA plates after adding imidacloprid, buprofezin, and abamectin at the low dose rate was 2.64 × 10^7^ ± 1.08 × 10^6^, 2.34 × 10^7^ ± 2.54 × 10^6^, and 1.65 × 10^7^ ± 9.92 × 10^5^, respectively; these were significantly different (F _2,8_ = 8.96, *p* = 0.02 < 0.05). The spore production of *M. flavoviride* at the medium dose rate of imidacloprid, buprofezin, and abamectin was 2.29 × 10^7^ ± 5.14 × 10^5^, 1.89 × 10^7^ ± 1.33 × 10^6^, and 1.44 × 10^7^ ± 8.32 × 10^5^, respectively; these were significantly different (F_2,8_ = 19.92, *p* = 0.0022 < 0.01). The spore production of *M. flavoviride* at the high dose rate of imidacloprid, buprofezin, and abamectin was 1.90 × 10^7^ ± 1.57 × 10^6^, 1.77 × 10^7^ ± 1.26 × 10^6^, and 1.34 × 10^7^ ± 2.95 × 10^5^, respectively; these were significantly different (F_2,8_ = 6.25, *p* = 0.0341 < 0.05).

### 3.3. Bioassays

#### 3.3.1. Effect of Different Bioassays on Mortalities with Adults and Second Instar Larvae of WFT

**Figure 3:**
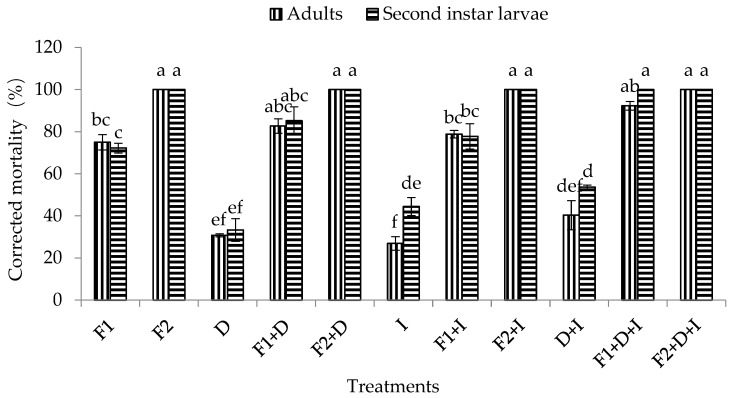
Effect of conidia concentrations of *M. flavoviride* associated with DE and imidacloprid on the mortality of adults and second instar larvae of WFT (*F. occidentalis*). Bars with the same letter above the bar are not significantly different (Tukey’s HSD at *p* = 0.05). Error bars denote SE values. D = DE at 200 ppm, F1 = *M. flavoviride* at 1.2 × 10^6^ conidia/mL, F2 = *M. flavoviride* at 1.2 × 10^8^ conidia/mL, I = imidacloprid at 50 ppm, D + I = DE at 200 ppm + imidacloprid at 50 ppm, D + F1 = DE at 200 ppm + *M. flavoviride* at 1.2 × 10^6^ conidia/mL, D + F2 = DE at 200 ppm + *M. flavoviride* at 1.2 × 10^8^ conidia/mL, I + F1 = imidacloprid at 50 ppm + *M. flavoviride* at 1.2 × 10^6^ conidia/mL, I + F2 = imidacloprid at 50 ppm + *M. flavoviride* at 1.2 × 10^8^ conidia/mL, D + I + F1 = DE at 200 ppm + imidacloprid at 50 ppm + *M. flavoviride* at 1.2 × 10^6^ conidia/mL, D + I +F2 = DE at 200 ppm + imidacloprid at 50 ppm + *M. flavoviride* at 1.2 × 10^8^ conidia/mL.

It was observed that DE, imidacloprid, and the combination of DE and imidacloprid had certain pathogenicity in the adults and second instar larvae of WFT, with corrected mortality rates in adults of 30.77%, 26.92%, and 40.38%, respectively, and corrected mortality rates in second instar larvae of 33.33%, 44.44%, and 53.70%, respectively. Moreover, the corrected mortality of the second instar larvae of WFT under the combined treatment with DE and imidacloprid was significantly higher than that of adults (F_1,5_ = 122.18, *p* = 0.0004 < 0.01) (Figure 3).

The mortality of adults and second instar larvae of WFT, in response to various conidia concentrations of the *M. flavoviride* conidia alone (assay 1); together with DE at 200 ppm (assay 2); with imidacloprid at 50 ppm (assay 3); or with DE and imidacloprid (assay 4) after 10 days of inoculation are illustrated in Figure 3. The mortality of adults and second instar larvae of WFT observed in the four different bioassays were concentration- and time-dependent, regardless of whether *M. flavoviride* was used alone or in combination with DE or imidacloprid (or even the fungus in combination with DE and imidacloprid). Furthermore, the corrected mortality under the four different bioassays increased with conidial concentrations. The final corrected mortality rates of adults and second instar larvae of WFT after 10 days of inoculation ranged from 75–100% and 72.22–100% in assay 1; 82.69–100% and 85.19–100% in assay 2; 78.85–100% and 77.77–100% in assay 3; and 92.31–100% and 100% in assay 4 (combination of *M. flavoviride* with DE and imidacloprid) (Figure 3). At a low concentration (1.2 × 10^6^ conidia/mL) of *M. flavoviride*, the corrected mortality rates of adults and second instar larvae of WFT were 75% and 72.22% in assay 1 (*M. flavoviride* alone); 82.69% and 85.19% in assay 2 (*M. flavoviride* with DE); 78.85% and 77.77% in assay 3 (*M. flavoviride* with imidacloprid); and 92.31% and 100% in assay 4 (*M. flavoviride* with DE and imidacloprid). The results showed that the mortalities were all significantly different from that in assay 4 (Adults: F_3,11_ = 6.74, *p* = 0.01 < 0.05; Larvae: F_3,11_ = 6.70, *p* = 0.01 < 0.05). However, at a high concentration (1.2 × 10^8^ conidia/mL) of *M. flavoviride*, the corrected mortality rates of adults and second instar larvae of WFT in assays 1 (*M. flavoviride* alone), 2 (*M. flavoviride* with DE), and 3 (*M. flavoviride* with imidacloprid) were not significantly different from that in assay 4 (*M. flavoviride* with DE and imidacloprid).

#### 3.3.2. Fitted TCM Relationships

The observed responses of the adults and second instar larvae of WFT from assays 1–4 fitted the TCM model well, with an accepted homogeneity fit based on the Hosmer–Lemeshow statistic *C* (*p* ≥ 0.05) (Table 2 and Table 3), to the adults and second instar larvae of WFT: (*C* = 3.73, df = 8, *p* = 0.88 for assay 1; *C* = 4.79, df = 8, *p* = 0.78 for assay 2; *C* = 12.38, df = 8, *p* = 0.14 for assay 3; *C* = 11.96, df = 8, *p* = 0.15 for assay 4) and (*C* = 2.26, df = 8, *p* = 0.97 for assay 1; *C* = 4.67, df = 7, *p* = 0.70 for assay 2; *C* = 15.49, df = 8, *p* = 0.05 for assay 3; *C* = 7.11, df = 8, *p* = 0.53 for assay 4), respectively. In addition, the *t*-statistics of all time-effect parameters estimated were significant (*p* < 0.0001); that is, the standard error was extremely small relative to the parameter estimates, indicating that the dose-effect and time-effect in the test strain *M. flavoviride* were extremely significant.

The estimated parameters (*β*) for the effect of *M. flavoviride* concentration were 0.53, 0.57, 0.92, and 1.06 for the adults, and 0.46, 0.46, 1.04, and 0.64 for the second instar larvae of WFT in assay 1 (*M. flavoviride* alone), assay 2 (*M. flavoviride* with DE), assay 3 (*M. flavoviride* with imidacloprid), and assay 4 (*M. flavoviride* with DE and imidacloprid), respectively; which indicated that the pathogenicity of *M. flavoviride* integrated with DE and imidacloprid against adult WFT (assay 4) was greater than those in assays 1–3, and that the pathogenicity of *M. flavoviride* integrated with imidacloprid against the second instar larvae of WFT (assay 3) was greater than that of assays 1, 2, and 4. This result further indicates that *M. flavoviride* combined with DE and imidacloprid exerted significantly greater control efficacy against WFT adults; however, *M. flavoviride* combined with imidacloprid exerted significantly greater control efficacy against the second instar larvae of WFT. The parameters for the conditional time effects (*γ_j_*) peaked at *γ*_6_ for the WFT adults, indicating that the highest mortality was at 6 days after treatment in assay 1. The real maximum mortality peaked at 4 and 8 days, and 4 and 7 days after treatment in assays 2 and 3, respectively. In assay 4, the highest mortality peaked at 7 days after treatment. However, the parameters for the conditional time effects (*γ_j_*) of the second instar larvae of WFT reached the highest mortality at 6 and 8 days after the inoculation treatment in assay 1. The true highest mortality peaks were obtained at 6, and 3 and 7 days after inoculation treatment in assays 2 and 3, respectively. In assay 4, the peak real highest mortality was achieved at 7, 9, and 10 days after inoculation treatment.

The fitted parameters γ_j_ were different between the developmental stages of *F. occidentalis*, with differences among the bioassay methods. This indicates that the time-specific effects and biocontrol potential of *M. flavoviride* at the tested conidial concentrations also varied with the different bioassay methods.

#### 3.3.3. Lethal Concentrations (LC_50_ and LC_90_) of *M. flavoviride* against the Adults and Second Instar Larvae of WFT under Different Bioassays

**Figure 4:**
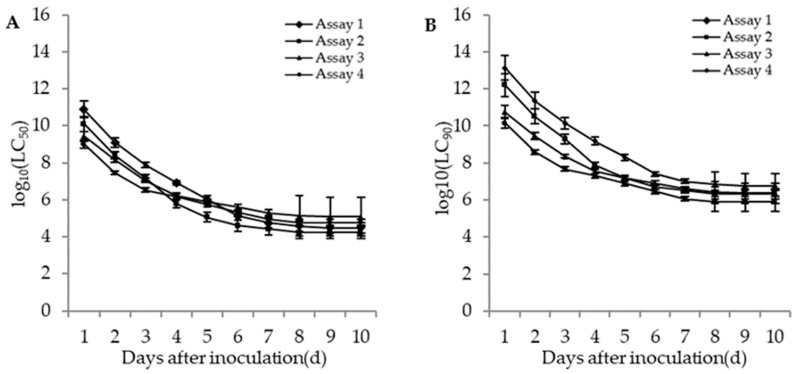
Curve trends in the logarithmic estimates of LC_50_ (**A**) and LC_90_ (**B**) (bars: SE) for assays 1 (*M. flavoviride* applied at 1.2 × 10^6^ and 1.2 × 10^8^ conidia/mL alone), 2 (*M. flavoviride* applied at 1.2 × 10^6^ and 1.2 × 10^8^ conidia/mL integrated with DE), 3 (*M. flavoviride* applied at 1.2 × 10^6^ and 1.2 × 10^8^ conidia/mL integrated with imidacloprid), and 4 (*M. flavoviride* applied at 1.2 × 10^6^ and 1.2 × 10^8^ conidia/mL integrated with DE and imidacloprid) against the adults of WFT (*F*. *occidentalis*) against days after inoculation.

**Figure 5:**
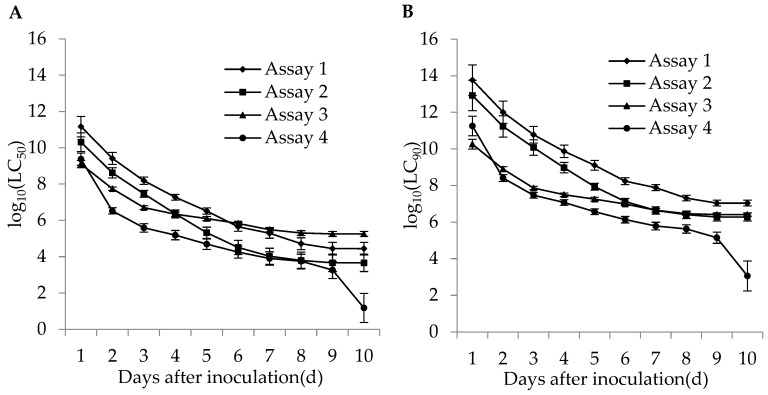
Curve trends in the logarithmic estimates of LC_50_ (**A**) and LC_90_ (**B**) (bars: SE) for assays 1 (*M. flavoviride* applied at 1.2 × 10^6^ and 1.2 × 10^8^ conidia/mL alone), 2 (*M. flavoviride* applied at 1.2 × 10^6^ and 1.2 × 10^8^ conidia/mL integrated with DE), 3 (*M. flavoviride* applied at 1.2 × 10^6^ and 1.2 × 10^8^ conidia/mL integrated with imidacloprid), and 4 (*M. flavoviride* applied at 1.2 × 10^6^ and 1.2 × 10^8^ conidia/mL integrated with DE and imidacloprid) against the second instar larvae of WFT (*F*. *occidentalis*) against days after inoculation.

The lethal concentrations (LC_50_ and LC_90_) of the adults and second instar larvae of WFT were estimated based on the TCM model in different bioassays. The lethal concentrations in different bioassays for the adults and second instar larvae of WFT decreased with an increase of incubation time (Figure 4 and Figure 5). The more insecticides (imidacloprid and DE) that were contained in the fungal concentrations, the lower the estimated LC_50_. For the adult WFT, the estimated LC_50_ values between days 3 and 10 in the four bioassays were 7.56 × 10^7^–3.06 × 10^4^ conidia/mL in assay 1, 1.58 × 10^7^–1.73 × 10^4^ conidia/mL in assay 2, 1.13 × 10^7^–1.20 × 10^5^ conidia/mL in assay 3, and 3.39 × 10^6^–6.07 × 10^4^ conidia/mL in assay 4; further, the LC_90_ values in the four bioassays were estimated to be 1.40 × 10^10^–5.65 × 10^6^ conidia/mL in assay 1, 2.00 × 10^9^–2.19 × 10^6^ conidia/mL in assay 2, 2.31 × 10^8^–2.46 × 10^6^ conidia/mL in assay 3, and 4.61 × 10^7^–8.27 × 10^5^ conidia/mL in assay 4. For the second instar larvae WFT, between days 3 and 10, the LC_50_ values in the four bioassays were estimated to be 1.53 × 10^8^–2.79 × 10^4^ conidia/mL in assay 1, 2.90 × 10^7^–4.61 × 10^3^ conidia/mL in assay 2, 5.06 × 10^6^–1.80 × 10^5^ conidia/mL in assay 3, and 3.88 × 10^5^–15.01 conidia/mL in assay 4; similarly, the LC_90_ values in the four bioassays were estimated to be 5.99 × 10^10^–1.09 × 10^7^ conidia/mL in assay 1, 1.19 × 10^10^–1.90 × 10^6^ conidia/mL in assay 2, 7.28 × 10^7^–2.59 × 10^6^ conidia/mL in assay 3, and 2.95 × 10^7^–1.14 × 10^3^ conidia/mL in assay 4.

In conclusion, the estimated LC_50_ and LC_90_ values for the adults and second instar larvae of WFT in assay 4 (*M. flavoviride* with DE and imidacloprid) were lower than those in assays 1 (*M. flavoviride* alone), 2 (*M. flavoviride* with DE), and 3 (*M. flavoviride* with imidacloprid). It was also shown that the pathogenicity of *M. flavoviride* integrated with DE and imidacloprid on the adults and second instar larvae of WFT was higher than those of *M. flavoviride* alone, *M. flavoviride* with DE, and *M. flavoviride* with imidacloprid. Obviously, the lethal concentration trends highlight the dependence of the fungus, DE, and insecticide interactions on post-inoculation time.

#### 3.3.4. Lethal Times (LT_50_ and LT_90_) of *M. flavoviride* against the Adults and Second Instar Larvae of WFT under Different Bioassays

The LT_50_ values were estimated by interpolation in the fitted TCM model of each bioassay method, which decreased with increasing *M. flavoviride* conidial concentration. This decrease (at a given fungal level) was significantly intensified by the combination of imidacloprid or DE (Table 4).

For adults and second instar larvae of WFT, both LT_50_ and LT_90_ among assays 1–4 decreased with increasing fungal conidial concentration. The TCM model predicted the LT_50_ and LT_90_ values for high conidia concentrations in assays 1–4; however, for assays 1, 2, and 3 at lower conidia concentrations (1.2 × 10^6^ conidia/mL), the LT_90_ could not be predicted as the mortality rate under them was less than 90%. For adults and second instar larvae of WFT, the lowest LT_50_ and LT_90_ values were estimated at the higher conidia concentration (1.2 × 10^8^ conidia/mL) in assay 4 (*M. flavoviride* with DE and imidacloprid), which were 1.53 and 2.69 d, and 1.41 and 2.45 d, respectively. For adult WFT, the LT_50_ values were 2.82, 2.26, 2.06, and 1.53 d at the higher conidia concentration (1.2 × 10^8^ conidia/mL) in assays 1–4, respectively. Similarly, for the second instar larvae of WFT, the LT_50_ values at the higher conidia concentration (1.2 × 10^8^ conidia/mL) in assays 1–4 were 3.11, 2.45, 1.70, and 1.41 d, respectively. The above results show that the virulence of assay 1 (*M. flavoviride* alone) was lower than that in assays 2 (*M. flavoviride* with DE), 3 (*M. flavoviride* with imidacloprid), and 4 (*M. flavoviride* with DE and imidacloprid), which further indicates that the entomopathogenic fungus combined with imidacloprid or DE could improve the control effect on adults and second instar larvae of WFT.

### 3.4. Conidia Production from WFT Carcasses

Mycelium and conidia of *M. flavoviride* WSWL51721 were detected on WFT carcasses by fluorescence microscopy. Then, 20 adults and the second instar larvae of WFT were selected from the four different bioassays. The sporulated *M. flavoviride* on adults and second instar larvae were cultured in petri dishes lined with moist, sterilized filter paper for 12 days.

The average number of newly produced conidia found on infected WFT adults after incubation varied over the ranges (3.63 ± 0.18) × 10^5^–(5.28 ± 0.13) × 10^5^ conidia/adult treated with *M. flavoviride* alone (assay 1); (3.32 ± 0.04) × 10^5^–(4.62 ± 0.04) × 10^5^ conidia/adult treated with *M. flavoviride* plus DE (assay 2); (3.57 ± 0.19) × 10^5^–(4.87 ± 0.12) × 10^5^ conidia/adult treated with *M. flavoviride* plus imidacloprid (assay 3); and (3.60 ± 0.32) × 10^5^–(5.17 ± 0.22) × 10^5^ conidia/adult treated with *M. flavoviride* plus DE and imidacloprid (assay 4), with a significant effect of the different bioassays on overall new post-mortem conidiogenesis (F_7,23_ = 20.70, *p* < 0.01). Similarly, the mean number of newly produced conidia found on infected second instar larvae of WFT after incubation varied over the ranges (2.60 ± 0.17) × 10^5^–(4.10 ± 0.35) × 10^5^ conidia/larva treated with *M. flavoviride* alone (assay 1); (2.50 ± 0.06) × 10^5^–(3.67 ± 0.09) × 10^5^ conidia/larva treated with *M. flavoviride* plus DE (assay 2); (2.87 ± 0.19) × 10^5^–(3.73 ± 0.09) × 10^5^ conidia/larva treated with *M. flavoviride* plus imidacloprid (assay 3); and (2.77 ± 0.09) × 10^5^–(3.50 ± 0.06) × 10^5^ conidia/larva treated with *M. flavoviride* plus DE and imidacloprid (assay 4), with a highly significant effect of the different bioassays on quantitative conidiogenesis (F_7,23_ = 13.38, *p* < 0.01) (Table 5).

## 4. Discussion

Several studies on integrated pest management (IPM) have focused on the pathogenicity of *B. bassiana* and *M. anisopliae*, as well as the combination of *B. bassiana* with predatory mites or different insecticides against WFT [9,10,13,53]. However, there have been no studies on the effects of different combinations of EPF with adjuvants and compatible insecticides on WFT. In addition, no fungal candidate of *M. flavoviride* which has a good control effect on the WFT has been found. In our previous studies, it was found from many different fungal candidates that *M. flavoviride* WSWL51721 has good potential in controlling WFT adults, in which the mortality reached 82.65% at a concentration of 1.0 × 10^7^ conidia/mL [26]. The compatibility levels between different chemical insecticides and EPF have been shown to be different [19]. We chose three chemical insecticides—imidacloprid, buprofezin, and abamectin—that have been commonly used to control WFT in the field. The influence of these three insecticides on the radial growth and conidial production rates of *M. flavoviride* were tested. The results showed that imidacloprid had good compatibility with *M. flavoviride*. Therefore, we conducted further studies to evaluate the efficacy of different combinations of *M. flavoviride* with DE and imidacloprid against adults and second instar larvae of WFT under laboratory conditions.

As a natural insecticide, DE has been applied to control many stored grain insect pests, achieving a good control effect [32,33,34,35,36]. However, there have been few reports on its use in the control of agricultural pests. Our results showed that DE has a certain control effect on the invasive insect WFT, which is similar to its control effect against invasive red fire ants, whose efficacy was lower than stored grain insect pests [32,35,37,38]. The differences in control effects were due to the fact that some insects are more sensitive to DE, due to their anatomical and physiological characteristics, and may also be related to differences in the cuticular composition of different insect species [34,37].

In this study, we found that the corrected mortalities of WFT adults and second instar larvae were 75% and 72.22%, respectively, at 10 days after inoculation of 1.2 × 10^6^ conidia/mL in assay 1 (*M. flavoviride* alone); while the corrected mortality of WFT adults and second instar larvae was 100% at 10 days after inoculation with 1.2 × 10^8^ conidia/mL, which is significantly higher than the results obtained for other pathogenic fungi of WFT [6,13,20,24]. However, the corrected mortalities of WFT adults and second instar larvae in assays 2 (*M. flavoviride* with DE), 3 (*M. flavoviride* with imidacloprid), and 4 (*M. flavoviride* with DE and imidacloprid) were higher than those in assay 1 (*M. flavoviride* alone), indicating that EPF combined with DE (assay 2), EPF combined with insecticide (assay 3), and EPF combined with DE and insecticide (assay 4) all had synergistic effects, thereby increasing the pathogenicity of EPF to WFT. These results were consistent with the studies reported by Athanassiou et al., in which a six-fold increase in adult mortality of *T. confusum* was observed when treated with *B. bassiana* and DE, compared with either *B. bassiana* or DE alone [15]. Similarly, Ashraf et al. clearly indicated that *M. anisopliae* combined with DE or thiamethoxam had a synergistic effect, compared to their individual efficacies, and was more effective in controlling four stored insect pests [35]. Likewise, Wakil et al. found that *B. bassiana* combined with DE or the neonicotinoid insecticide thiamethoxam increased the mortality of *R. dominica* adults, compared with individual treatments, and a significant additive effect was observed [32]. Similar synergistic interactions were observed when the tested fungi *B. bassiana*, *M. anisopliae*, and *I. fumosorosea* were combined with DE against *Plodia interpunctella*, *Ephestia cautella*, and *Ephestia kuehniella* [51]. Therefore, it is important to understand how a pathogen interacts with other insecticides and adjuvants. However, at present, little is known about the reasons for such synergism; it is generally believed that DE has unique physical properties: When it contacts insects, it will destroy the wax layer of the insect cuticles and cause water exudation from the body, which can provide favorable conditions for the attachment and germination of conidia, which can quickly penetrate the cuticle layer of the host insect body wall and then into the haemocoel, continuing to multiply and secrete a series of metabolites which, finally, leads to mycosis and death [39,40,41,42]. For neonicotinoid insecticides such as imidacloprid and thiamethoxam, when the target insect comes into contact with it, normal conduction of the central nervous system will be blocked, leading to rapid paralysis before death; this can assist the conidia in penetrating the weakened insects more easily. The fungi will eventually kill the weakened insect relatively quickly.

In time–concentration–response tests, the TCM modeling method has been applied in fungi–insect [60,61,62,63,64] and insecticide–insect bioassays [65,66,67]. In this study, the lethal times and concentrations in different bioassays were evaluated by inserting experimental data into the TCM model. We found that the lethal concentrations (LC_50_ and LC_90_) and lethal times (LT_50_ and LT_90_) for adult WFT treated with *M. flavoviride* plus DE and imidacloprid (assay 4) were lower than those of other bioassay methods (i.e., assays 1, 2, and 3), while the lethal concentrations and the lethal times of WFT second instar larvae in assay 4 were also lower than those of the bioassay methods (assays 1, 2, and 3). These results are in accordance with the above-mentioned results: the mortality of WFT adults and second instar larvae in assay 4 was higher than in the other bioassays (assays 1, 2, and 3), indicating that different combinations of EPF with DE and imidacloprid have different synergistic effects. Similarly, Kivett et al. clearly found that *M. anisopliae* combined with azadirachtin had synergistic effects on WFT larvae [6].

The carcasses caused by the infection of fungal agents play a very important role in the secondary recycling of infection, as the conidia produced on mycotized carcasses can be spread to other sites, which can infect the target insects and other pests, increasing the effective persistency of the fungal biocontrol agents [39,55,68]. Information on the post-mortem sporulation of carcasses after killing WFT by EPF had not been reported. In our study, we found that there was no significant difference in the sporulation of the carcasses between the four different bioassays of the WFT adults, indicating that DE and imidacloprid had no clear effect on the quantitative conidiogenesis of dead WFT adults. This may be due to the minimal amounts of imidacloprid and DE remaining on the surface of the WFT adults after inoculation, as well as passive or active removal of adjuvants on the cuticle by grooming and decomposition behaviors [55]. However, there was a significant difference in the sporulation of dead WFT second instar larvae in four different bioassays. We speculate that this may be related to the different components of the cuticle of WFT in different developmental stages.

## 5. Conclusions

In conclusion, our study showed that *M. flavoviride* WSWL5172 was not only highly virulent to adult WFT, but also to second instar larvae WFT under laboratory conditions. Most importantly, the combination of the fungal strain *M. flavoviride* WSWL5172 with DE and imidacloprid had a good control effect on the adult and second instar larvae of WFT, where the lethal time was decreased by half, compared with that of *M. flavoviride* WSWL5172 alone. Considering that the efficacy of EPF in the field is not as good as that indoors (as, in the field, EPF are affected by environmental factors, such as temperature, humidity, and light [17]), the combination of chemical insecticides or adjuvants with EPF will become the trend of IPM programs in future.

## Figures and Tables

**Table 1:** Combinations of *Metarhizium flavoviride*, diatomaceous earth, and imidacloprid against the adults and second instar larvae of WFT (*F. occidentalis*) in efficacy studies.

Assay	*Metarhizium flavoviride* (conidia/mL)	Diatomaceous Earth (ppm)	Imidacloprid (ppm)
A 1	1.2 × 10^6^	0	0
	1.2 × 10^8^	0	0
A 2	1.2 × 10^6^	50	0
	1.2 × 10^8^	50	0
A 3	1.2 × 10^6^	0	200
	1.2 × 10^8^	0	200
A 4	1.2 × 10^6^	50	200
	1.2 × 10^8^	50	200

**Table 2:** Virulence parameters of *M. flavoviride* WSWL51721 against adult WFT.

Method	Conditional Mortality Model				Cumulative Mortality Model			
	Parameter ^a^	Value	S.E.	*t* ^b^	Parameter ^a^	Value	var (*τ_j_*)	cov (*β,τ_j_*)
Assay 1	*β*	0.53	0.11	4.94	*β*	0.53	0.00	0.00
	*γ*_1_	−6.13	0.86	7.16	*τ*_1_	−6.13	0.19	−0.02
	*γ*_2_	−5.68	0.84	6.78	*τ*_2_	−5.19	0.18	−0.02
	*γ*_3_	−5.28	0.83	6.38	*τ*_3_	−4.54	0.17	−0.02
	*γ*_4_	−4.95	0.82	6.04	*τ*_4_	−4.03	0.17	−0.02
	*γ*_5_	−4.54	0.80	5.68	*τ*_5_	−3.56	0.16	−0.02
	*γ*_6_	−4.06	0.77	5.30	*τ*_6_	−3.09	0.15	−0.02
	*γ*_7_	−4.57	0.82	5.58	*τ*_7_	−2.88	0.15	−0.02
	*γ*_8_	−5.23	0.98	5.37	*τ*_8_	−2.79	0.15	−0.02
	*γ*_9_	−5.82	1.28	4.54	*τ*_9_	−2.74	0.14	−0.02
	*γ*_10_	−15.01	0.00	1501224821.11	*τ*_10_	−2.74	0.14	−0.02
*H-L* test ^c^	*C*_8_ = 3.73, *p* = 0.88							
Assay 2	*β*	0.57	0.11	5.08	*β*	0.57	0.01	0.01
	*γ*_1_	−6.13	0.88	6.98	*τ*_1_	−6.13	0.28	−0.04
	*γ*_2_	−5.65	0.87	6.52	*τ*_2_	−5.17	0.27	−0.04
	*γ*_3_	−5.17	0.86	6.04	*τ*_3_	−4.48	0.26	−0.04
	*γ*_4_	−4.26	0.81	5.24	*τ*_4_	−3.67	0.25	−0.03
	*γ*_5_	−4.35	0.78	5.61	*τ*_5_	−3.26	0.23	−0.03
	*γ*_6_	−4.44	0.80	5.57	*τ*_6_	−2.99	0.22	−0.03
	*γ*_7_	−5.26	1.00	5.26	*τ*_7_	−2.89	0.21	−0.03
	*γ*_8_	−5.08	1.04	4.91	*τ*_8_	−2.79	0.21	−0.03
	*γ*_9_	−15.26	0.00	1526174665.79	*τ*_9_	−2.77	0.21	−0.03
	*γ*_10_	−15.26	0.00	1526170593.00	*τ*_10_	−2.79	0.21	−0.03
*H-L* test ^c^	*C*_8_ = 4.79, *p* = 0.78							
Assay 3	*β*	0.92	0.14	6.78	*β*	0.92	0.01	0.01
	*γ*_1_	−9.01	1.07	8.41	*τ*_1_	−9.01	0.44	−0.05
	*γ*_2_	−8.20	1.06	7.74	*τ*_2_	−7.84	0.42	−0.05
	*γ*_3_	−7.29	1.04	7.03	*τ*_3_	−6.83	0.41	−0.05
	*γ*_4_	−6.72	0.92	7.27	*τ*_4_	−6.08	0.36	−0.05
	*γ*_5_	−7.04	0.91	7.78	*τ*_5_	−5.75	0.33	−0.05
	*γ*_6_	−6.95	0.97	7.20	*τ*_6_	−5.49	0.32	−0.05
	*γ*_7_	−6.66	0.97	6.91	*τ*_7_	−5.22	0.31	−0.05
	*γ*_8_	−7.07	11.72	0.60	*τ*_8_	−5.07	1.26	−0.05
	*γ*_9_	−8.01	1.37	5.86	*τ*_9_	−5.02	1.17	−0.05
	*γ*_10_	−17.36	0.00	1736162872.06	*τ*_10_	−5.02	1.17	−0.05
*H-L* test ^c^	*C*_8_ = 12.38, *p* = 0.14							
Assay 4	*β*	1.06	0.15	7.12	*β*	1.06	0.01	0.01
	*γ*_1_	−9.90	1.17	8.43	*τ*_1_	−9.90	0.63	−0.08
	*γ*_2_	−8.47	1.16	7.31	*τ*_2_	−8.25	0.61	−0.08
	*γ*_3_	−7.76	1.04	7.45	*τ*_3_	−7.28	0.53	−0.07
	*γ*_4_	−8.08	0.98	8.25	*τ*_4_	−6.91	0.48	−0.07
	*γ*_5_	−7.46	0.96	7.78	*τ*_5_	−6.45	0.45	−0.07
	*γ*_6_	−7.00	0.96	7.29	*τ*_6_	−6.00	0.42	−0.07
	*γ*_7_	−6.69	0.98	6.80	*τ*_7_	−5.59	0.41	−0.06
	*γ*_8_	−7.34	1.17	6.26	*τ*_8_	−5.43	0.41	−0.06
	*γ*_9_	−18.23	0.00	1822796044.24	*τ*_9_	−5.43	0.41	−0.06
	*γ*_10_	−18.23	0.00	1822790822.24	*τ*_10_	−5.43	0.41	−0.06
*H-L* test ^c^	*C*_8_ = 11.96, *p* = 0.15							

^a^ The subscript of γ represents the specific day after inoculation. ^b^ The t-statistics for all the parameter estimates were highly significant (*p* < 0.0001). ^c^ *H-L* test: Homogeneity hypothesis for the goodness of fit was accepted when *p* ≥ 0.05 in the Hosmer–Lemeshow test (with the degrees of freedom denoted by the subscript of *C*). Assay 1: *M. flavoviride* was applied at 1.2 × 10^6^ and 1.2 × 10^8^ conidia/mL alone against the adults of WFT (*F*. *occidentalis*). Assay 2: *M. flavoviride* was applied at 1.2 × 10^6^ and 1.2 × 10^8^ conidia/mL integrated with DE against the adults of WFT. Assay 3: *M. flavoviride* was applied at 1.2 × 10^6^ and 1.2 × 10^8^ conidia/mL integrated with imidacloprid against the adults of WFT. Assay 4: *M. flavoviride* was applied at 1.2 × 10^6^ and 1.2 × 10^8^ conidia/mL integrated with DE and imidacloprid against the adults of WFT.

**Table 3:** Virulence parameters of *M. flavoviride* WSWL51721 against WFT second instar larvae.

Method	Conditional Mortality Model				Cumulative Mortality Model			
	Parameter ^a^	Value	S.E.	*t* ^b^	Parameter ^a^	Value	var (*τ_j_*)	cov (*β,τ_j_*)
Assay 1	*β*	0.46	0.10	4.48	*β*	0.46	0.00	0.00
	*γ*_1_	−5.54	0.82	6.73	*τ*_1_	−5.54	0.20	−0.02
	*γ*_2_	−5.31	0.81	6.53	*τ*_2_	−4.73	0.19	−0.02
	*γ*_3_	−4.99	0.80	6.22	*τ*_3_	−4.16	0.18	−0.02
	*γ*_4_	−4.81	0.80	5.99	*τ*_4_	−3.74	0.18	−0.02
	*γ*_5_	−4.60	0.79	5.86	*τ*_5_	−3.38	0.18	−0.02
	*γ*_6_	−4.08	0.78	5.27	*τ*_6_	−2.98	0.17	−0.02
	*γ*_7_	−4.70	0.82	5.71	*τ*_7_	−2.82	0.17	−0.02
	*γ*_8_	−4.01	0.80	5.01	*τ*_8_	−2.55	0.17	−0.02
	*γ*_9_	−4.56	0.89	5.10	*τ*_9_	−2.43	0.16	−0.02
	*γ*_10_	−14.61	0.00	1460589375.39	*τ*_10_	−2.43	0.16	−0.02
*H-L* test ^c^	*C*_8_ = 2.26, *p* = 0.97							
Assay 2	*β*	0.46	0.10	4.43	*β*	0.46	0.00	0.00
	*γ*_1_	−5.10	0.81	6.33	*τ*_1_	−5.10	0.25	−0.03
	*γ*_2_	−4.94	0.81	6.11	*τ*_2_	−4.32	0.24	−0.03
	*γ*_3_	−4.68	0.80	5.88	*τ*_3_	−3.79	0.24	−0.03
	*γ*_4_	−4.21	0.78	5.40	*τ*_4_	−3.29	0.23	−0.03
	*γ*_5_	−3.78	0.76	4.95	*τ*_5_	−2.81	0.22	−0.03
	*γ*_6_	−3.61	0.75	4.80	*τ*_6_	−2.44	0.21	−0.03
	*γ*_7_	−3.82	0.81	4.71	*τ*_7_	−2.21	0.21	−0.03
	*γ*_8_	−4.40	0.97	4.56	*τ*_8_	−2.11	0.20	−0.03
	*γ*_9_	−4.93	1.21	4.07	*τ*_9_	−2.05	0.20	−0.03
	*γ*_10_	−14.58	0.00	1458324483.35	*τ*_10_	−2.05	0.20	−0.03
*H-L* test ^c^	*C*_7_ = 4.67, *p* = 0.70							
Assay 3	*β*	1.04	0.14	7.36	*β*	1.04	0.01	0.01
	*γ*_1_	−9.80	1.12	8.78	*τ*_1_	−9.80	0.48	−0.06
	*γ*_2_	−8.68	1.12	7.78	*τ*_2_	−8.40	0.48	−0.06
	*γ*_3_	−7.73	1.04	7.46	*τ*_3_	−7.32	0.43	−0.06
	*γ*_4_	−8.09	0.98	8.28	*τ*_4_	−6.94	0.40	−0.06
	*γ*_5_	−8.20	0.97	8.48	*τ*_5_	−6.69	0.37	−0.05
	*γ*_6_	−7.84	0.95	8.26	*τ*_6_	−6.41	0.35	−0.05
	*γ*_7_	−7.26	0.93	7.82	*τ*_7_	−6.06	0.34	−0.05
	*γ*_8_	−7.62	0.99	7.67	*τ*_8_	−5.87	0.33	−0.05
	*γ*_9_	−8.83	1.37	6.46	*τ*_9_	−5.81	0.33	−0.05
	*γ*_10_	−18.09	0.00	1809361422.42	*τ*_10_	−5.81	0.33	−0.05
*H-L* test ^c^	*C*_8_ = 15.49, *p* = 0.05							
Assay 4	*β*	0.64	0.12	5.45	*β*	0.64	0.01	0.01
	*γ*_1_	−6.35	0.91	7.00	*τ*_1_	−6.35	0.39	−0.05
	*γ*_2_	−4.71	0.87	5.39	*τ*_2_	−4.53	0.36	0.05
	*γ*_3_	−4.73	0.82	5.80	*τ*_3_	−3.93	0.33	−0.05
	*γ*_4_	−5.17	0.82	6.30	*τ*_4_	−3.68	0.31	−0.04
	*γ*_5_	−4.66	0.81	5.76	*τ*_5_	−3.36	0.30	−0.04
	*γ*_6_	−4.50	0.85	5.32	*τ*_6_	−3.08	0.29	−0.04
	*γ*_7_	−4.46	0.96	4.66	*τ*_7_	−2.86	0.28	−0.04
	*γ*_8_	−5.13	1.29	3.97	*τ*_8_	−2.76	0.28	−0.04
	*γ*_9_	−3.79	1.06	3.59	*τ*_9_	−2.45	0.29	−0.04
	*γ*_10_	−1.42	0.00	142261625.14	*τ*_10_	−1.12	0.28	−0.01
*H-L* test ^c^	*C*_8_ = 7.11, *p* = 0.53							

^a^ The subscript of γ represents the specific day after inoculation. ^b^ The t-statistics for all the parameter estimates were highly significant (*p* < 0.0001). ^c^ *H-L* test: Homogeneity hypothesis for the goodness of fit was accepted when *p* ≥ 0.05 in the Hosmer–Lemeshow test (with the degrees of freedom denoted as the subscript of *C*). Assay 1: *M. flavoviride* was applied at 1.2 × 10^6^ and 1.2 × 10^8^ conidia/mL alone against the second instar larvae of WFT (*F*. *occidentalis*). Assay 2: *M. flavoviride* was applied at 1.2 × 10^6^ and 1.2 × 10^8^ conidia/mL integrated with DE against the second instar larvae of WFT. Assay 3: *M. flavoviride* was applied at 1.2 × 10^6^ and 1.2 × 10^8^ conidia/mL integrated with imidacloprid against the second instar larvae of WFT. Assay 4: *M. flavoviride* was applied at 1.2 × 10^6^ and 1.2 × 10^8^ conidia/mL integrated with DE and imidacloprid against the second instar larvae of WFT.

**Table 4:** Lethal time values (LT_50_ and LT_90_) for the adults and second instar larvae of WFT infected by *M. flavoviride* WSWL51721 in four different bioassays.

Developmental Stages	LT_50_/LT_90_ (d)	Bioassay and Conidia Concentration (conidia/mL)							
		Assay 1		Assay 2		Assay 3		Assay 4	
		1.2 × 10^6^	1.2 × 10^8^	1.2 × 10^6^	1.2 × 10^8^	1.2 × 10^6^	1.2 × 10^8^	1.2 × 10^6^	1.2 × 10^8^
Adults	LT_50_	4.94	2.82	3.77	2.26	4.42	2.06	4.23	1.53
	LT_90_	—	5.31	—	3.90	—	3.48	6.98	2.69
Second instar larvae	LT_50_	5.49	3.11	4.26	2.45	5.07	1.70	2.45	1.41
	LT_90_	—	6.47	—	4.89	—	2.86	6.18	2.45

Note: Dashes denote values that could not be predicted under the different inoculation conidia concentration by the TCM model. Assay 1: *M. flavoviride* applied at 1.2 × 10^6^ and 1.2 × 10^8^ conidia/mL alone. Assay 2: *M. flavoviride* applied at 1.2 × 10^6^ and 1.2 × 10^8^ conidia/mL integrated with DE. Assay 3: *M. flavoviride* applied at 1.2 × 10^6^ and 1.2 × 10^8^ conidia/mL integrated with imidacloprid. Assay 4: *M. flavoviride* applied at 1.2 × 10^6^ and 1.2 × 10^8^ conidia/mL integrated with DE and imidacloprid.

**Table 5:** Conidia production from carcasses of adults and second instar larvae of WFT infected by *M. flavoviride* WSWL51721.

Heading	Treatments of Additive or Formulation	Number of Conidia/Adult × 10^5^	Number of Conidia/Second Instar Larva × 10^5^
T 1	F1	3.63 ± 0.18b	2.60 ± 0.17d
	F2	5.28 ± 0.13a	4.10 ± 0.35a
T 2	D + F1	3.32 ± 0.04b	2.50 ± 0.06d
	D + F2	4.62 ± 0.04a	3.67 ± 0.09ab
T 3	I + F1	3.57 ± 0.19b	2.87 ± 0.19bcd
	I + F2	4.87 ± 0.12a	3.73 ± 0.09a
T 4	D + I + F1	3.60 ± 0.32b	2.77 ± 0.09cd
	D + I + F2	5.17 ± 0.22a	3.50 ± 0.06abc
		*F*_7,23_ 20.70	*F*_7,23_ 13.38
		*p* = 0.001 < 0.01	*p* = 0.001 < 0.01

Within each insect developmental state, means followed by the same letter are not significantly different (Tukey’s HSD at *p* = 0.05) (bars: SE). D = DE at 200 ppm, F1 = *M. flavoviride* at 1.2 × 10^6^ conidia/mL, F2 = *M. flavoviride* at 1.2 × 10^8^ conidia/mL, I = imidacloprid at 50 ppm, D + F1 = DE at 200 ppm + *M. flavoviride* at 1.2 × 10^6^ conidia/mL, D + F2 = DE at 200 ppm + *M. flavoviride* at 1.2 × 10^8^ conidia/mL, I + F1 = imidacloprid at 50 ppm + *M. flavoviride* at 1.2 × 10^6^ conidia/mL, I + F2 = imidacloprid at 50 ppm + *M. flavoviride* at 1.2 × 10^8^ conidia/mL, D + I + F1 = DE at 200 ppm + imidacloprid at 50 ppm + *M. flavoviride* at 1.2 × 10^6^ conidia/mL, D + I + F2 = DE at 200 ppm + imidacloprid at 50 ppm + *M. flavoviride* at 1.2 × 10^8^ conidia/mL.
